# Second Recurrence of a Mitotically Active Cellular Fibroma From the Ovary

**DOI:** 10.1097/og9.0000000000000004

**Published:** 2024-03-26

**Authors:** Casey Wilkerson, Emma Barr, Babac Shahmohamady, Alireza Abidi

**Affiliations:** Department of Obstetrics and Gynecology, Adventist Health White Memorial Medical Center, Los Angeles, and Obstetrics and Gynecology and Gynecologic Oncology, Kaiser Permanente Riverside Medical Center, Riverside, California.

## Abstract

**BACKGROUND:**

Mitotically active cellular fibroma is a benign fibromatous tumor of the ovary. It has 4 or more mitoses/10 high-power fields like a malignant sarcoma but has low-grade nuclear characteristics. Treatment is surgical excision, and recurrence is rare.

**CASE:**

We present a primary and secondary recurrence of mitotically active cellular fibroma. This patient was a 65-year-old woman who underwent a total abdominal hysterectomy and bilateral salpingo-oophorectomy for a large mitotically active cellular fibroma. She then had recurrences of the tumor 5 and 10 years later, requiring repeat resections.

**CONCLUSION:**

We recommend continued follow-up with these patients given the possibility for delayed recurrence and because follow-up and preventative treatment for these tumors are not well studied.

## Abstract 2

Benign mitotically active cellular fibromas of the ovary may be more aggressive than previously thought and can recur years after surgical resection.

**TEACHING POINTS**

1. Mitotically active cellular fibromas are benign ovarian tumors with an increased mitotic rate similar to a sarcoma.
2. Surgical resection of mitotically active cellular fibromas previously was thought to be curative; however, these benign tumors may recur in the years after removal. Therefore, continued follow-up is recommended.

Fibromas are rare ovarian tumors originating from sex cord-stomal tissue. They account for 4% of ovarian neoplasms and tend to be benign and curable with surgical excision.^1^ Rarely, these tumors are malignant and are known as *fibrosarcomas*. The fibrosarcoma has severe nuclear atypia and 4 or more mitoses/10 high-power fields (HPF) compared with the fibroma, which has bland nuclei and 3 or fewer mitoses/10 HPF.^2^ As defined by Prat and Scully in 1981, fibromatous tumors of the ovary were thought to fall into one of those two categories.^3^ However, in 2006, Irving et al^2^ published a case series reporting 40 cases of fibromas with increased mitotic rate but low-grade nuclear characteristics that had benign outcomes. They termed these tumors “mitotically active cellular fibromas”. These tumors had 4 or more mitoses/10 HPF like a fibrosarcoma; however, the nuclear atypia was absent. Follow-up was available for 18 of the 40 cases, ranging from 3 months to 12 years, and there were no recurrences after surgical excision. After this publication, mitotically active cellular fibroma was recognized in 2014 by the World Health Organization’s Classification of Tumors of Female Reproductive Organs as a new category of fibromatous tumor.^4^

## CASE

**Fig. 1.**
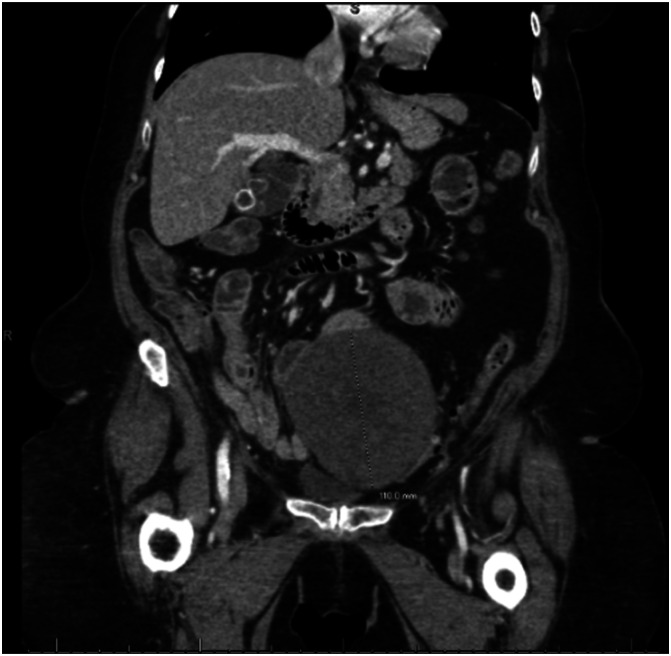
Computed tomography scan demonstrating the initial 14.6-cm pelvic mass in 2013.

A 65-year-old woman presented in 2013 with abdominal pain and distension. A computed tomography (CT) scan of the abdomen and pelvis demonstrated a 14.6-cm lobulated complex mass in the midline pelvis, abutting the superior aspect of the uterus (Fig. 1). The patient underwent exploratory laparotomy, total abdominal hysterectomy, bilateral salpingo-oophorectomy, and partial omentectomy. Intraoperative findings included a 17.5-cm solid right ovarian mass adherent to the uterus, right pelvic side wall, and large intestine. There were no ascites or other intraabdominal findings. Due to significant adhesions, a complete omentectomy was not performed. Frozen section revealed a spindle cell tumor with increased mitotic activity (9 mitoses/10 HPF). There was no significant nuclear atypia. Final pathology was a mitotically active cellular fibroma.

*Wilkerson. Recurrent Mitotically Active Cellular Fibroma. O&G Open 2024*.

**Fig. 2.**
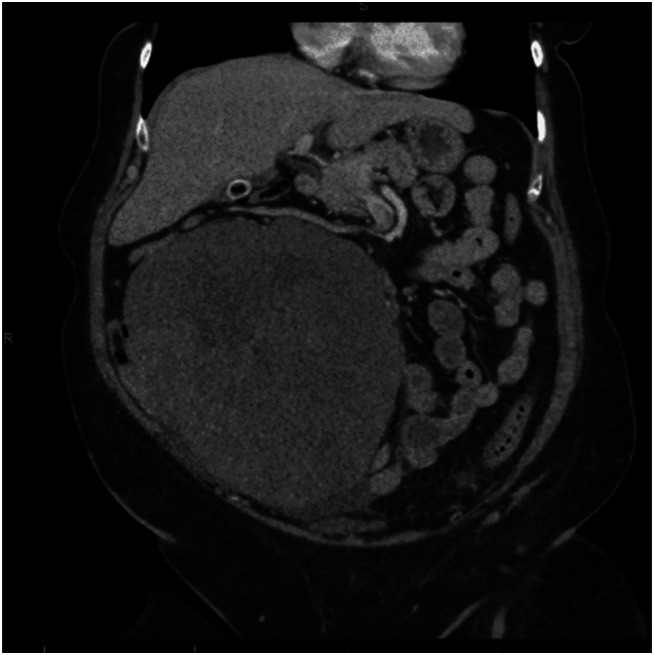
Computed tomography scan shows a 19.7-cm recurrent pelvic mass in 2018.

The patient followed up once in clinic but was not seen by a gynecologist again until she presented 5 years later, in 2018, with a palpable abdominal mass. A CT scan confirmed a 17.5×11.3×19.7–cm lobulated, heterogeneous soft tissue mass in the right abdomen, with extrinsic mass effect against decompressed loops of small bowel (Fig. 2). There was also a second heterogenous soft tissue mass in the left lower abdomen measuring 14.5×10.2×15 cm. Laparotomy demonstrated a 20.5-cm abdominal mass with multiple loops of densely and intimately adherent small bowel, including the jejunum and ileum. There was an additional 16.5-cm pelvic mass adherent to the cul de sac. The masses appeared to be arising from the bowel mesentery. Pelvic mass excision was performed. The small bowel was carefully dissected off the mass; however, a 10-cm portion of the ileum located 15 cm from the ileocecal valve was attached to the mass and was resected with primary reanastomosis. Pathology of both masses confirmed recurrent mitotically active cellular fibroma, similar to the primary ovarian tumor in 2013. There were 10 mitoses/10 HPF with no nuclear atypia. Immunostain for estrogen receptor was negative. Outside pathologic consultation was obtained, which confirmed mitotically active cellular fibroma. The patient was monitored postoperatively with quarterly in-office examinations and biannual imaging. However, she was lost to follow-up 1.5 years later during the coronavirus disease 2019 (COVID-19) pandemic.

*Wilkerson. Recurrent Mitotically Active Cellular Fibroma. O&G Open 2024*.

**Fig. 3.**
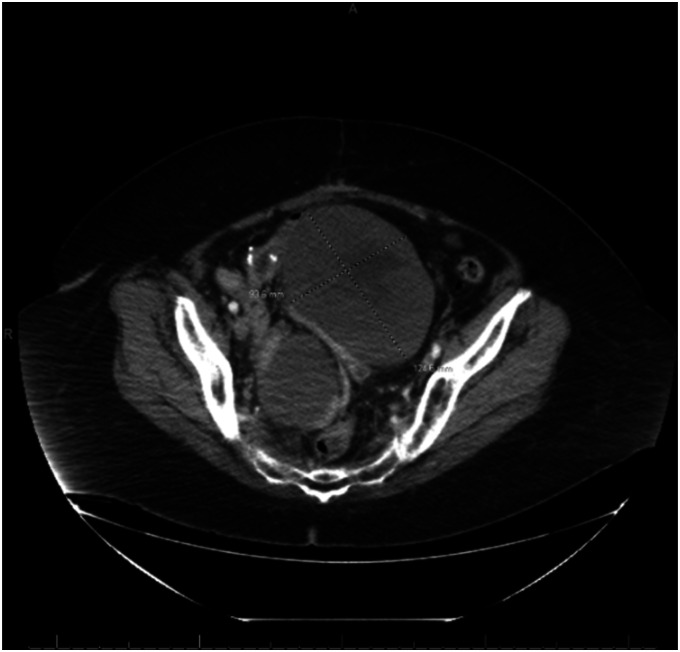
Two new pelvic masses, 12.5 cm and 9 cm, seen on computed tomography scan in 2023.

**Fig. 4.**
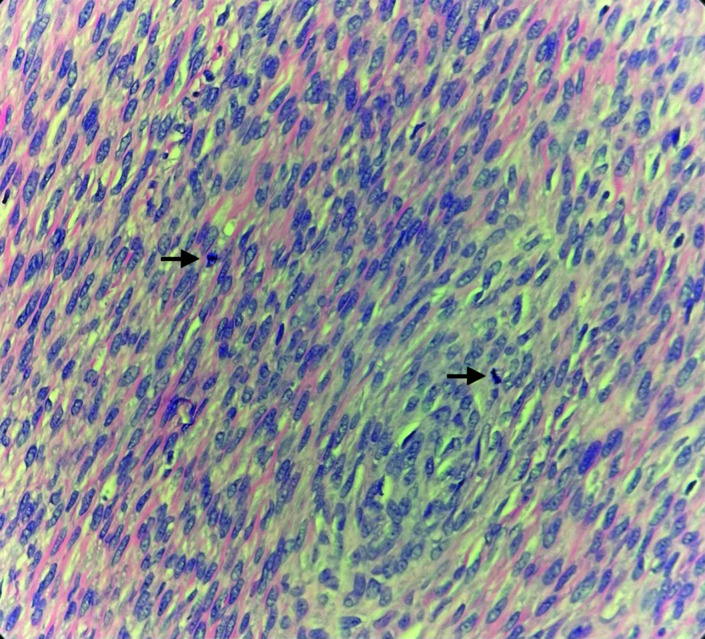
Hematoxylin and eosin stain at 40× magnification showing two mitoses (*arrows*) in one high-power field taken from one of the pelvic masses in 2023.

In 2023, 5 years after the second procedure, the patient was admitted to the hospital with nausea and vomiting. A CT scan confirmed two new solid pelvic masses measuring 12.5×9.4×11.0 cm and 9.1×6.0×8.4 cm (Fig. 3). A CT-guided biopsy confirmed a spindle cell neoplasm that was estrogen receptor/progesterone receptor–negative. The patient underwent a third exploratory laparotomy, which demonstrated three pelvic masses measuring 14 cm, 12 cm, and 5 cm that were densely adherent to the small bowel, with penetration to the mesentery causing de-vascularization of the small bowel and partial obstruction. Extensive lysis of adhesions was performed; however, a loop of small bowel was fused to the mass and was therefore removed en bloc with the mass. Additional small bowel was resected due to multiple enterotomies and areas of devascularization; 105 cm of small bowel and an intact ileo-cecal valve were preserved with side-to-side anastomosis. An implant also was seen on the remaining omentum and was sent for pathologic review. Final pathology of the masses and the omental tumor implant returned as recurrent mitotically active cellular fibroma with more than 10 mitoses/10 HPF (Fig. 4). The tumor was positive for WT-1, SF1, and CD10 and negative for inhibin, desmin, and smooth muscle actin. The patient did well postoperatively, with return of bowel function. She has started letrozole therapy and will be followed with imaging and serial examinations every 3 months for the first 2 years, then every 6 months.

*Wilkerson. Recurrent Mitotically Active Cellular Fibroma. O&G Open 2024*.

*Wilkerson. Recurrent Mitotically Active Cellular Fibroma. O&G Open 2024*.

## DISCUSSION

Since the World Health Organization recognized mitotically active cellular fibroma in 2014, multiple cases of recurrence have been published. A literature search was performed with PubMed and Google Scholar in August 2023. Search terms included mitotically active cellular fibroma and recurrent ovarian fibroma. All cases of recurrent mitotically active cellular fibroma were reviewed; there was no date or year limit. In 2021, Olivadese published a case report of a recurrence occurring 16 years after the initial unilateral salpingo-oophorectomy. This is thought to be the longest interval of mitotically active cellular fibroma recurrence. The original tumor had only 4 mitoses/10 HPF and recurred as a peritoneal nodule with only 2 mitoses/10 HPF. Immunostains were positive for vimentin, CD10, and estrogen and progesterone receptor. The patient was followed for 8 years after the first recurrence, with no evidence of disease.^5^

The same year, Dawoud published a case with a much more rapid recurrence, only 6 months after the initial excision. The patient initially presented with an 18-cm ovarian mass and underwent surgical excision by exploratory laparotomy, hysterectomy, bilateral salpingo-oophorectomy, and appendectomy. Surgery required a small bowel excision and reanastomosis for removal. The tumor had 17 mitoses/10 HPF, with a Ki-67 index of 50%. Ischemic necrosis and perforation were noted, but there was no evidence of tumor infiltrate in the bowel adhesions or pelvic washings. Six months later, the patient was found to have a new pelvic mass that was confirmed by CT-guided biopsy as mitotically active cellular fibroma. A second surgery was deferred because the patient had multiple significant medical comorbidities, making her a poor surgical candidate. She was treated conservatively with tamoxifen but died 2 months later.^6^

To our knowledge, aside from the case we present here, there is only one other published case of a second recurrence. Bucella et al reported a 65-year-old woman who presented with a 12-cm pelvic mass 5 years after undergoing total hysterectomy and bilateral salpingo-oophorectomy for a 10-cm ovarian fibroma. The patient underwent laparoscopic excision, and the mass was removed in a bag. The tumor cells were positive for vimentin but negative for CD10. The Ki-67 index was 9%, with 4 mitoses/10 HPF. There was no atypia or necrosis noted. Slides were reviewed from the primary tumor and found to be similar. The patient was therefore diagnosed with a recurrent mitotically active cellular fibroma. Six months later, the patient was found to have a new 8-cm pelvic mass on magnetic resonance imaging. The mass was excised by laparotomy and had the same pathologic findings as the first recurrence. The patient began treatment with tamoxifen, with the goal of preventing another recurrence. She was followed for 6 months before publication, with no new recurrences.^7^

We present a rare case of mitotically active cellular fibroma with not only one but two recurrences, each occurring 5 years after the prior excision. Additionally, at the time of both her second and third procedures, the patient was found to have multiple masses growing from the small bowel mesentery and even a small implant on the omentum. The initial tumor was removed grossly intact from the abdomen by laparotomy, with no mention of rupture or spill in the operative report. However, it was noted to be adherent to the pelvic sidewall and large bowel; therefore, microscopic disease still may have been present.

Follow-up and preventative treatment for these tumors are not well studied. Given that small bowel resection was required for removal in both recurrences, the patient is at high risk for morbidity and mortality should she have a third recurrence. She was started on letrozole after the second recurrence in an attempt to prevent another recurrence. Two of the cases described above also used hormone therapy, but with tamoxifen in lieu of surgical excision and to prevent additional recurrence postoperatively.^6,7^ However, long-term follow-up is not available. The tumors were not sent for next-generation sequencing; however, this could be considered in similar cases to evaluate for any genetic abnormalities.

Although these tumors were first described in 2006, there is still much to learn about their behavior and potential for recurrence. They are not defined as malignant pathologically, yet it seems that mitotically active cellular fibromas do have the ability to spread throughout the abdomen, with long-term sequelae.
